# NMDA Receptors in Astrocytes: In Search for Roles in Neurotransmission and Astrocytic Homeostasis

**DOI:** 10.3390/ijms20020309

**Published:** 2019-01-14

**Authors:** Katarzyna Skowrońska, Marta Obara-Michlewska, Magdalena Zielińska, Jan Albrecht

**Affiliations:** Department of Neurotoxicology, Mossakowski Medical Research Centre, Polish Academy of Sciences, 5 Pawińskiego Str., 02-106 Warsaw, Poland; martomich@gmail.com (M.O.-M.); mzielinska@imdik.pan.pl (M.Z.)

**Keywords:** astrocyte, NMDA receptor, glutamine synthetase, aquaporin 4, Kir4.1, neuroprotection, neurotransmission

## Abstract

Studies of the last two decades have demonstrated the presence in astrocytic cell membranes of *N*-methyl-d-aspartate (NMDA) receptors (NMDARs), albeit their apparently low abundance makes demonstration of their presence and function more difficult than of other glutamate (Glu) receptor classes residing in astrocytes. Activation of astrocytic NMDARs directly in brain slices and in acutely isolated or cultured astrocytes evokes intracellular calcium increase, by mutually unexclusive ionotropic and metabotropic mechanisms. However, other than one report on the contribution of astrocyte-located NMDARs to astrocyte-dependent modulation of presynaptic strength in the hippocampus, there is no sound evidence for the significant role of astrocytic NMDARs in astrocytic-neuronal interaction in neurotransmission, as yet. Durable exposure of astrocytic and neuronal co-cultures to NMDA has been reported to upregulate astrocytic synthesis of glutathione, and in this way to increase the antioxidative capacity of neurons. On the other hand, overexposure to NMDA decreases, by an as yet unknown mechanism, the ability of cultured astrocytes to express glutamine synthetase (GS), aquaporin-4 (AQP4), and the inward rectifying potassium channel Kir4.1, the three astroglia-specific proteins critical for homeostatic function of astrocytes. The beneficial or detrimental effects of astrocytic NMDAR stimulation revealed in the in vitro studies remain to be proven in the in vivo setting.

## 1. Introduction

Research of the last few decades has promoted astrocytes from cells providing physical and metabolic support to neurons to being their indispensable partner in neurotransmission (for exhaustive reviews see [1,2]). One of the crucial tools which astrocytes use to communicate with neurons and to modulate their function are neurotransmitter receptors located on astrocytic cell membranes. Indeed, astrocytes are endowed with a rich repertoire of receptors for neuroactive amino acids, purines, and catecholamines, among these receptors recognizing glutamate (Glu), the major excitatory neurotransmitter in the central nervous system (CNS). Like in neurons, Glu receptors present on astrocytes include metabotropic glutamate receptors (mGluR) and the three classes of ionotropic receptors (iGluR): α-amino-3-hydroxy-5-methyl-4-isoxazolepropionic acid (AMPA) receptors (AMPAR), kainic acid (KA) receptors (KAR), and *N*-methyl-d-aspartate receptors (NMDARs) [3,4,5]. Of these, the status of NMDARs is relatively least explored and has long been considered uncertain. Pioneering studies of the 1980s, which analysed electrophysiological and calcium flux-generating responses of astrocytes to Glu in primary cell culture preparations, failed to demonstrate the presence of functional NMDARs on astrocytes. While the responses to Glu could be mimicked by KA or the AMPAR agonist quisqualate, it was not the case with NMDA or Glu in the presence of NMDAR antagonists [6,7,8,9,10]. The advent of experiments on acutely isolated brain slices led first to detailed characterisation of astrocytic AMPAR [11], the responses of which to Glu differed between astrocytes (i) in brain slices and in culture [12], and (ii) recorded in two different loci of the same brain region (hippocampus) [13]. NMDA-mediated responses to Glu were first successfully recorded in astrocytes in brain slices and in astrocytes derived from the brain by the fluorescence-activated cell sorting (FACS) technique [14], albeit interference of the direct response of astrocytes to NMDA by an indirect effect mediated by Glu transfer between astrocytes and neurons could not be excluded in this study. A study performed in acutely isolated astrocytes rendered the first description of NMDAR-mediated currents evoked in astrocytes by exogenously applied Glu [15]. From then on, one could witness considerable progress in understanding the nature of NMDARs residing in astrocytes, including their region-to-region and sub-regional heterogeneity with regard to their pharmacological and functional properties and feedback response to adjacent neurons. Independently, intensive work has led to the revival of cultured astrocytes as a valuable tool with which to study intracellular effects of stimulation of astrocytic NMDARs, including modulation of the responses by pathogenic events. This review accounts for hallmarks of the progress, but at the same time addresses questions that remain to be unraveled to appreciate how critical the role of these receptors is in CNS metabolism and functions. Three major issues will be in focus:

1. Subunit composition and assembly of NMDAR in astrocytes and the impact of the NMDAR composition on receptor functionality.
2. Ways in which astrocytic NMDARs respond to extrasynaptic Glu and translate the neuron-derived signal to modulation of neurotransmission.
3. The role of signals elicited by astrocytic NMDARs in regulating astroglia-specific intracellular metabolic events and in shaping the response of astrocytes to pathogenic stimuli.

Inconsistencies of the results obtained using different ex vivo and in vitro experimental settings and inherent limitations of the methodology used so far will be emphasised.

## 2. Subunit Composition and Properties of NMDAR in Astrocytes: Comparison to Neurons

Most of the current knowledge about the structure and functioning of NMDAR is derived from studies on neurons. Investigations on neurons revealed the existence of seven NMDAR subunits which form hetero-tetrameric transmembrane ion channels. They contain two GluN1 (In many earlier articles, GluN1, GluN2, and GluN3 have been designated interchangeably with NR1, NR2, and NR3. In this review, we will be exclusively using the terms GluN1, GluN2, and GluN3, which is now a generally accepted mode) subunits, which bind the co-agonist [glycine (Gly) or d-serine] and are linked to one or two GluN2 (A,B,C,D) subunits and one GluN3 (A,B) subunit. GluN2 are Glu-binding subunits, while GluN3 also binds Gly (or d-serine) [16,17]. GluN1 is the obligatory NMDAR subunit essential for the assembly and trafficking of other subunits, and for the formation of a functional NMDAR channel [18,19,20]. GluN3 acts in a dominant-negative manner to suppress receptor activity [21,22]. Overexpression of GluN3A protein results in the formation of GluN1/GluN3A channels that offer neuroprotection [23]. 

The neuronal NMDARs are classically composed of two GluN1 and two GluN2A or GluN2B. Two molecules of the co-agonist Gly [24,25] and two molecules of the agonist Glu are required for their activation ([26,27,28,29,30]; see [31] for a recent review). More rarely detected NMDAR variants include:

- assemblies of GluN1 with GluN2C/D or GluN3A/B,
- a heterodimer of GluN1 and GluN3A/B subunits that forms an excitatory Gly receptor (interaction with Gly is sufficient for their activation) [32,33].
- a complex composed of GluN1/GluN2/GluN3 subunits that form an NMDAR producing lower currents compared to GluN1/GluN2 assemblies ([32,34,35,36,37], reviewed by [38]).

Each receptor subtype by itself exhibits temporal and regional specificity [39,40], and their unique functional properties are determined by the specific combination of subunits [41,42]. The subunit composition-dependent receptor properties include Glu affinity, receptor desensitisation, ion conductance, agonist affinity, sensitivity to allosteric modulation, and pharmacological sensitivity [27,38,42]. NMDARs possessing GluN2C/D subunits are less sensitive to Mg^2+^ block than those composed of GluN2A/B, and the combination of GluN3 subunit with GluN2 and GluN1 additionally reduces the sensitivity to Mg^2+^ and limits Ca^2+^ permeability [16,21,27,32,33,37,38,43]. Specifically, the presence of GluN3A subunit is critical for determination of the Mg^2+^ block and Ca^2+^ permeability of NMDARs [21,44,45,46]. 

Expression of NMDAR in astrocytes has been fairly reproducibly documented at the level of mRNA coding for the different subunits. Messenger RNAs for GluN1, GluN2A, and GluN2B subunits have been detected in cultured mouse [47] and rat [48] astrocytes. Moreover, transcripts for the GluN1, GluN2B, and GluN2C NMDAR subunits have been demonstrated in cortical astrocytes [14] and in Bergmann glia [49]. In mouse astrocytes in vivo, the presence of all NMDAR subunits at different expression levels was demonstrated [50], with GluN1, GluN2C, and GluN3A being the quantitatively dominating subunits [51,52,53,54]. Transcripts for all seven NMDAR subunits (i.e., GluN1, GluN2A-D, and GluN3A,B) have been found in cultured human [55] and rat astrocytes [56]. 

With regard to subunit proteins, immunohistochemical staining of brain slices combined with electron microscopic analysis demonstrated the expression in astrocytes from different rat brain regions of GluN1 [57,58,59,60,61] and GluN2A/B [57,60]. Moreover, the expression of GluN2C protein was documented in astrocytes from Grin2C^tm1 (EGFP/cre/ERT2)Wtsi^ mouse line [62]. A full set of 7 subunits was recorded in cultured rat [56] and human astrocytes [55]. The GluN1 subunit was detected by immunostaining in mouse cultured astrocytes [47], whereas GluN2B was detected in both mouse and rat astrocytes in vitro [47,63].

At the protein level (Western Blot method), so far only the expression of GluN1 [56], GluN2A, and GluN2B [48] subunits have been demonstrated in rat astrocytes in culture.

NMDARs expressed in astrocytes differ from neuronal NMDARs in their weak susceptibility of the channel to Mg^2+^ blockade (about −120mV) and reduced Ca^2+^ permeability (*P*_Ca_/*P*_monovalent_ ~3). The difference is most likely due to the relative overrepresentation of the tri-heteromeric (GluN1 + GluN2 + GluN3) configuration of NMDAR in astrocytes [15,31,38,52,64,65,66,67]. In one of the above-mentioned studies, functional NMDAR composed of GluN1 with one GluN2C or GluN2D subunit and one GluN3 subunit were identified in astrocytes [66]. Most recently, predominant expression of mRNA coding for the GluN2C subunit was reported in glial cells of the telencephalon [68]. In most studies, requirement of astrocytic NMDAR activity for the co-agonists glycine or d-serine has been reported ([15,52,66,69,70], however, see [56] for a contradictory observation).

## 3. Changes in Astrocytic NMDAR Subunit Expression in Brain Pathologies

**Figure 1:**
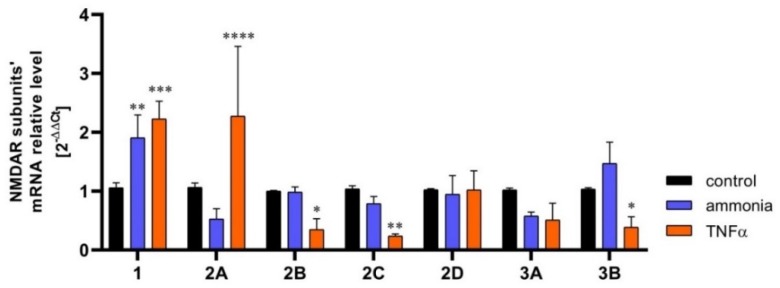
The content of mRNA coding for NMDAR subunits in mouse cortical astrocytes cultured in control conditions and exposed for 72 h to 5 mM ammonia or 50 ng/ml TNFα treatment. Real-time PCR procedure was exactly as in Skowrońska et al., (2019) [72]. The mRNA expression was determined by TaqMan Gene Expression Assay (Applied Biosystems). The assay IDs were Mm00433790_m1 for GluN1 (Grin1); Mm00433802_m1 for GluN2A (Grin2a); Mm00433820_m1 for GluN2B (Grin2b); Mm00439180_m1 for GluN2C (Grin2c); Mm00433822_m1 for GluN2D (Grin2d); Mm01341723_m1 for GluN3A (Grin3a); Mm00504568_m1 for GluN3B (Grin3b); and Mm00607939_s1 for endogenous control β-actin. Results are mean ± SD (*n* = 3). (*) *p* < 0.05 vs control; (**) *p* < 0.01 vs control; (***) *p* < 0.001 vs control; (****) *p* < 0.0001 vs control; Two-Way ANOVA with Dunnett post-hoc test.

Krebs et al., (2003) [71] reported transient increase of the expression of the GluN1 and GluN2B NMDAR subunits in hippocampal astrocytes in a rat model of ischemia, induced by carotid artery clamping, but provided no mechanistic insight to this process. This study demonstrated a similar GluN2B overexpression in cultured hippocampal astrocytes in culture subjected to anoxia, corroborating the ex vivo studies. The response of astrocytic NMDARs to ischemia was further investigated by Zhou et al., (2010) [47]. The study, conducted on mouse cultured astrocytes, confirmed an increase in GluN2B expression modelled with oxygen-glucose deprivation (OGD). However, the expression dynamics of GluN1 were biphasic; initially it increased, and then decreased below the control level at 6h. The GluN2A expression decrease became apparent at 4 h and remained low up to the 6th h. The pathophysiological implications of the above changes were not commented on by the authors and remain to be envisaged. Dzamba et al., (2015) [52] confirmed the involvement of astrocytic NMDARs in the pathomechanism of ischemia. They observed increased GluN3A subunit expression in astrocytes from the cerebral cortex of mice subjected to permanent middle cerebral artery occlusion (MCAo). The astrocytes of ischemic animals also had impaired (diminished) Ca^2+^ response to NMDA as compared to sham-operated controls. Experiments from our own laboratory revealed changes in the expression of mRNA coding in a number of NMDAR subunits in astrocytes exposed to two neurotoxic factors: ammonia and the inflammatory cytokine TNFα (Figure 1; for exhaustive characterisations of these neurotoxins see Section 7).

## 4. Mechanisms and Manifestations of NMDAR Activation in Astrocytes 

**Figure 2:**
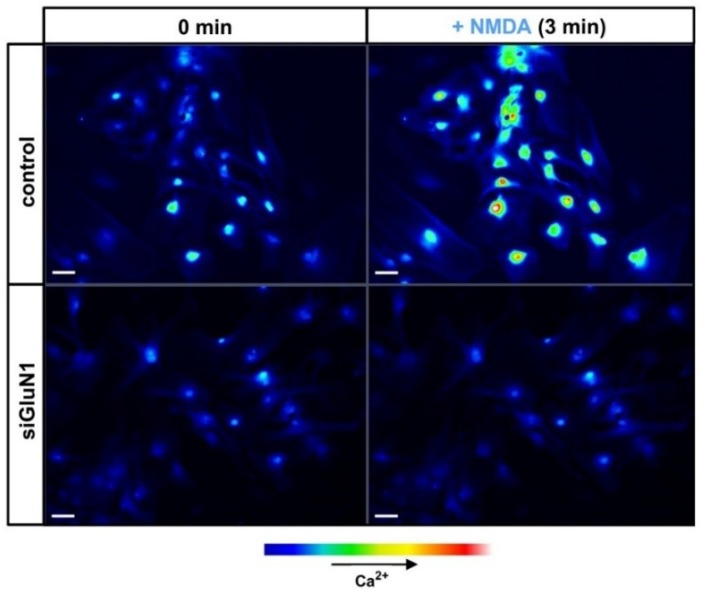
Confocal images of [Ca^2+^] changes in cultured mouse astrocytes before (control) and after silencing GluN1 subunit of NMDAR (siGluN1). Silencing procedure was exactly as in Skowrońska et al., (2019) [72]. Cells were pre-loaded with the fluorescent Ca^2+^ indicator, Fluo-3-AM. The images display untreated cells (0 min, left) and cells treated with 100 μM NMDA (3 min, right). The strength of the [Ca^2+^] signal is expressed by the relative intensity of Fluo-3-AM fluorescence in a pseudo-color scale (bottom: pseudo-color bar). Scale bars, 50 μm.

Extensive research of the last two decades has confirmed that astrocytes are able to respond to neuronal stimuli mediated by a variety of neurotransmitters, including Glu, and to modulate neuronal activity (reviewed in [1,2]). Unlike the metabotropic glutamatergic signalling in astrocytes, functionality of astrocytic NMDAR remains a matter of controversy due to the virtual lack of in situ experiments that would unequivocally confirm their role in CNS function. However, there is growing evidence from in vitro and ex vivo studies confirming that activation of NMDAR in astrocytes by Glu or selective NMDAR agonists mediates ion currents and intracellular calcium waves. NMDAR-evoked intracellular calcium accumulation in mouse cortical astrocytes before and after silencing of GluN1 with siRNA is illustrated in Figure 2.

NMDAR is a cationic channel with partial permeability for Ca^2+^. Accordingly, there has long been a consensus that influx from the extracellular space is the only mechanism by which stimulation of NMDAR increases intracellular Ca^2+^. The exclusivity of the ionotropic mechanism has been questioned in recent studies. In CA1 pyramidal neurons, in the presence of amyloid β (Aβ), NMDARs act as metabotropic receptors and activate intracellular signalling cascades in the absence of Ca^2+^ influx [73]. Such external Ca^2+^ flow-independent (metabotropic) NMDAR activity is also required for Aβ-induced synaptic depression [74,75,76]. In acute hippocampal slices, activation of NMDARs induced long-term depression (LTD) without ion flow through the receptors ([77,78,79,80], see [81] for a recent review). As will be described below, astrocytic NMDARs have likewise been observed to act through non-canonical, metabotropic signalling pathways. 

Zhang et al., (2003) [82] and Hu et al., (2004) [83] have noted that in rat astrocytes, calcium increase, as a response to Glu, NMDA, or AMPA, was partially inhibited by the NMDAR antagonist: 2-amino-5-phosphonopentanoate (AP5) and AMPAR/KAR antagonist 6-cyano-7-nitroquinoxaline-2,3-dione (CNQX), indicating both NMDAR and AMPAR-dependence. While the sensitivity to lack of external Ca^2+^ supported the involvement of an ionotropic mechanism, the inhibition of Glu-induced Ca^2+^ flux by the endoplasmic reticulum (ER) SERCA ATPase blocker, thapsigargin, indicated that metabotropic response is involved as well. Similar conclusions could be drawn from a recent study by Montes de Oca Balderas and Aguilera (2015) [56]. In their hands, the NMDA-induced calcium entry to astrocytes was sensitive to NMDAR antagonists AP5 and kynurenic acid (KYNA) and was blocked by siRNA knockdown of the GluN1 subunit, supporting the ionotropic mechanism. However, the response turned out to be also sensitive to the antagonists of ryanodine and IP3 receptors, ryanodine, and xestospongin C, but not to the NMDAR channel blocker MK-801 or the absence of calcium in the medium. Furthermore, the group revealed that NMDAR activity is dependent on tyrosine kinase function (inhibition of the kinase by genistein potentiated the NMDA-induced calcium signal). In cultured human astrocytes (cerebral white matter biopsies from tumour margin), Nishizaki et al., (1999) [70] and subsequently Kondoh et al., (2001) [69] have detected two types of NMDA-induced ion currents, mediated both by iGluR (insensitive to GDPβS, a broad G-protein inhibitor, and sensitive to external calcium depletion) and mGluR (AP5 independent). The NMDA-induced currents were enhanced by ~40% by 5 μM glycine. Similar evidence for the concurrence of the ionotropic and metabotropic mechanism of the NMDAR activity has also been reported in cultured rat astrocytes subjected to an inflammatory stimulus [63]. The physiological meaning of the dual mechanism remains to be elucidated. As a prerequisite, the duality must be proven in the in vivo setting. As a note of caution, it is not certain whether the metabotropic mechanism operates in all experimental contexts or preparations. In human foetal and adult cultured astrocytes, stimulation of intracellular Ca^2+^ accumulation by selective NMDAR agonists quinolinic acid (QUIN) and trans-ACBD was virtually abolished by NMDAR channel blockers memantine or MK-801 [55]. 

While Ca^2+^ influx is a commonly used marker of NMDAR activity in astrocytes, in one case known to us, a different marker was employed. ATP release from astrocytes, which is regarded as a major pathway employed in glia-neuron interaction, termed gliotransmission [84], is mainly triggered by activation of glutamatergic or purinergic metabotropic receptors [1]. However, as early as 1997, the group of von Kügelgen showed that NMDA (100 μM) induces ATP release in rat cortical astrocytes in culture [85]. The effect was blocked by a selective NMDAR antagonist, AP5, and was abolished or greatly reduced in the absence of external calcium [85,86]. Investigation of this interesting phenomenon has so far not been continued by others. 

The activity of NMDAR residing on astrocytes have been also verified and confirmed in different preparations of brain slices. In their pioneering work on acutely isolated rat spinal cord slices, in which cell membrane currents were recorded using the whole-cell patch-clamp technique, Ziak et al., (1998) [87] established that astrocytic NMDARs are characterised by lack of Mg^2+^ blockade and inhibition in the absence of external Ca^2+^. Schipke et al., (2001) [14] showed NMDA-induced inward ion currents in mouse cortical astrocytes selectively expressing an enhanced green fluorescence protein (EGFP). The currents were almost completely and irreversibly blocked by MK-801. Moreover, using microinjected fluorescent calcium dye Fluo-4-AM, the authors showed an increase in intracellular calcium, evoked by NMDA application. Both the ion currents and Ca^2+^ response were dependent on neuronal function, as demonstrated by tetradotoxin (TTX) sensitivity. In a similar experimental setting, Lalo et al., (2006) [15] confirmed the ability of NMDA to induce ion currents in astrocytes. They also showed that Glu evokes two types of current response, resulting from iGluR and Glu transporters, respectively. The iGluR involved were mostly NMDARs, and to much lesser extent, AMPAR. Additionally, the analysis of astrocytic NMDAR properties performed by Lalo et al., (2006) [15] revealed that NMDA-mediated currents are potentiated by the co-agonist glycine, but unlike in neuronal NMDAR, are insensitive to magnesium blockade. Moreover, astrocytes exhibited spontaneous miniature currents, both NMDA- and AMPA-mediated, in the presence of TTX or picrotoxin. The miniature currents were sensitive to NMDAR antagonists but not to the Glu transporter inhibitor, dl-threo-β-benzyloxyaspartate (TBOA). The presence of miniature potentials bespeaks local, direct neuron-glia communication. Palygin et al., (2011) [66] recorded NMDA-evoked, glycine and d-serine dependent (weak agonism) ionic currents in astrocytes analysed either directly in slices or following their acute isolation from mouse cerebral cortex. The responses were reduced by NMDAR antagonists UBP141, memantine and MK-801, but not by one other antagonist, ifenprodil. The Ca^2+^ permeability of astrocytic NMDAR was lower than of the neuronal NMDAR, and the Mg^2+^ block was absent. As mentioned earlier, such response pattern was a good fit into the model of NMDAR in murine cortical astrocytes, being a tri-heteromer assembled of not only GluN1 and GluN2 but also GluN3, a subunit responsible for both relatively low Ca^2+^ permeability and lack of Mg^2+^ block. As is to be recalled from Section 2, the presence of GluN3 in astrocytic NMDAR has been confirmed in different astrocytic preparations.

## 5. Translation of Astrocytic NMDAR Activation to Modulation of Neuronal Function

Research conducted so far has documented that modulation of neurotransmission by astrocytes occurs by means of the release of various gliotransmitters, and that the release mainly takes place in response to activation of two classes of receptors—the mGluR or the purinergic [1]. It is by now clear that activation of NMDARs in astrocytes generates intracellular calcium signalling. The question arose of whether this signal modulates the function of single neurons or neuronal networks. Up to this date, only one single report has appeared demonstrating coordination of synaptic events by astrocytic NMDARs.

Lettelier et al., (2016) [88] studied this issue ex vivo in mice hippocampal slices and in vitro in dissociated hippocampal cultures. In slices, the authors classically stimulated Schaffer collaterals, recorded excitatory postsynaptic currents (EPSC), and evaluated paired-pulse ratios in CA1 and CA3 regions of the hippocampus. To unequivocally confirm the involvement of astrocytic NMDAR, they used an animal model with a conditional knock-out of astrocytic NMDAR or selective, opto-genetically inducible hyperpolarisation of astrocytes (archaerhodopsin proton pump, ArchT), both obtained by a viral vector delivery. They also blocked pertinent NMDAR-activated processes with combinations of an astrocytic tricarboxylic acid (TCA) cycle inhibitor (fluoroacetate), NMDAR antagonist (AP5), and L-type voltage-gated Ca^2+^ channels (L-VGCCs) blockers (nifedipine, verapamil, QX-314). By those means, NMDAR involvement in a process of hetero-synaptic presynaptic plasticity has been established. The biological purpose of this type of plasticity is to exert an inhibitory tone on excitatory synapses. Such modulation of the postsynaptic response is executed by enhancing the diversity of synaptic strengths reaching the synapse. In detail, the following sequence has been proposed: Glu released from the active synapse (with high probability) activates NMDAR on the astrocyte enveloping the synapse, causes local depolarisation (L-VGCCs opening) and inducing calcium release within the astrocyte. The calcium signalling induces inhibitory gliotransmitter (e.g., ATP metabolised to adenosine or endocannabinoids) release strengthens the inhibitory tone on excitatory synapses, whereas locally, the high probability synapses are preferentially protected from the inhibition.

## 6. The Responses of Astroglia-Specific Intracellular Events to Persistent Stimulation of NMDAR in Astrocytes

The hitherto discussed evidence of astrocytic NMDAR function pertains to transient activation of NMDAR, which is to be considered as physiological response to neuronal activity involved in the maintenance of ambient conditions for proper neuronal firing. Knowledge of the effects of persistent stimulation of NMDAR in astrocytes, which is likely to occur in brain pathologies associated with an excitotoxic component, only begins to emerge.

The research of Jiang et al., (2010) [89] implies the engagement of astrocytic NMDAR in nitric oxide (NO) metabolism and synthesis. A prolonged (18 h) incubation of cultured rat cortical astrocytes with NMDA induced MK-801-sensitive translocation to nucleus of a carboxyl-terminal PDZ ligand of neuronal nitric oxide synthase (nNOS) (CAPON), a molecule that regulates nNOS stability, localisation, and expression during synapse formation.

A neuroprotective response of astrocytes subjected to 8 h treatment with NMDA was reported by Jimenez-Blasco et al., (2015) [48]. In this study, stimulation with NMDA of individual astrocytes derived from astrocytic-neuronal co-cultures resulted in calcium release from astrocytic ER, pointing to a metabotropic mechanism of NMDAR activation. The downstream sequence of events commenced with activation of protein kinase Cδ. Active protein kinase Cδ promoted formation of p35/cyclin-dependent kinase-5 (Cdk5) complex that further mediated nuclear factor-erythroid 2-related factor-2 (Nrf2) translocation to the nucleus and induced the expression of antioxidant genes (glutamate-cysteine ligase, hemeoxygenase-1, NADPH quinone oxidorreductase-1) and synthesis of glutathione (GSH). The newly synthesised GSH was then transferred to the neurons, which normally have a limited ability to synthesize this antioxidant. The results are interpreted to mimic the mechanism by which astrocytes protect neurons against oxidative stress.

Studies in the authors’ laboratory revealed deleterious, excitotoxic-like response to astrocytic NMDAR activation. In two subsequent studies, we investigated the responses of three astroglia-specific proteins: the inwardly rectifying potassium channel Kir4.1, the water channel aquaporin-4 (AQP4), and glutamine synthetase (GS). Their function is critical for astrocytes to execute key homeostatic roles in CNS, namely potassium buffering, water transport, and glutamine synthesis, respectively. Obara-Michlewska et al., (2015) [90] showed that expression of potassium channel Kir4.1 in cultured rat astrocytes is dependent on NMDAR, as both Glu and NMDA, administered for 72 h, decreased mRNA and protein level of Kir4.1, whereby the effect of Glu was prevented by MK-801 but not by the mGluR antagonist, MTEP. Recently, Skowrońska et al., (2019) [72] reported that exposure of mouse cortical astrocytes in vitro to NMDA for 8 or 72 h decreased the expression of Kir4.1, AQP4, and both the expression and enzyme activity of GS. The NMDAR-dependence of this process was confirmed by the lack of effects of NMDA in astrocytes, in which GluN1 was silenced by siRNA. The ionotropic character of this astrocytic NMDAR-mediated response was supported by inhibition of the agonist effects in calcium-free media. 

## 7. Involvement of Astrocytic NMDAR in the Effects of Brain Pathogenic Stimuli

As already mentioned in a different context in Section 3, Zhou et al., (2010) [47] observed that OGD-induced nitric oxide synthase 1 (NOS1) expression was prevented by co-treatment with NMDAR antagonist, MK-801. The result implies that astrocytic NMDAR may contribute to the nitrosative-oxidative stress evoked by the excitotoxicity accompanying the ischemic insult. 

Inflammation is one other pathological condition, where involvement of astrocytic NMDAR was postulated. In a study by Wu et al., (2009) [91], in primary midbrain rat neuron-glia cultures treated with lipopolysaccharide (LPS), memantine exerted neurotrophic and neuroprotective effects on dopaminergic neurons. Those effects were astrocyte-dependent, since they were not present in neuronal cultures devoid of astrocytes. The study implies that astrocytic NMDAR, being the target for memantine, mediated the glial cell line-derived neurotrophic factor (GDNF) synthesis. Gerard and Hansson (2012) [63] showed that the amplitude of calcium response was significantly increased in astrocytes exposed to LPS for 24 h. Moreover, AP5 or ifenprodil prevented the LPS-induced intensification of the calcium flux. Those antagonists inhibited also the LPS-potentiated proinflammatory cytokine interleukin-1β (IL-1β) release.

In rat primary astrocytes, 12-h stimulation with LPS or TNFα resulted in increased expression of proinflammatory chemokines (CXCL10 and CCL20), which was prevented by memantine [92]. The authors linked NMDAR function with the synthesis of growth factors [insulin-like growth factor 1 (IGF-1) and bone morphogenetic protein (BMP-4)], as memantine or MK-801 blocked their release upon stimulation with NMDA or Glu. The same experimental paradigm led the authors to a conclusion that GluN1 transcription is dependent on NMDAR. However, the nature of the experimental design limits interpretation of the latter two results.

Ting et al., (2009) [93] stimulated cultured astrocytes, derived from human foetal tissue, with QUIN, an excitotoxic product of kynurenine metabolism in microglia, or macrophages implicated in psychiatric disorders and neurodegenerative processes in the brain. QUIN induced IL-1β expression and dose-dependently inhibited activity of GS, an enzyme whose expression and activity in cultured astrocytes was also found inhibited by prolonged stimulation with NMDA [72].

Astrocytic NMDAR overactivation has been recently linked to the pathomechanism of Alzheimer’s disease. Li et al., (2016) [94] found that Aβ increases the expression of GluN2A and GluN2B NMDAR subunits in hippocampal astrocytes grown in the presence of hippocampal neurons, but not in their absence. Further, the authors showed that pre-treatment of astrocytes with NMDAR antagonists MK-801 or CGP39551 before introducing them into the co-culture system with neurons partly prevented Aβ-induced loss of two synaptic proteins—the postsynaptic density protein 95 (PSD95) and synaptophysin. The synaptoprotective effect was mediated by astrocytic NMDAR-induced synthesis of a neurotrophin, nerve growth factor β (β-NGF).

Ammonia is a major pathogenic factor in a variety of brain pathologies associated with hyperammonemia, including hepatic encephalopathy, a neuropsychiatric disorder resulting from liver failure. Ammonia primarily affects astrocytes via different overlapping molecular pathways, involving oxidative-nitrosative stress, ionic imbalance, disruption of energy metabolism, and neuroinflammation (reviewed in: [95]). Recently, astrocytic NMDAR has been implicated in the mechanism of ammonia neurotoxicity. Lachmann et al., (2013) [96] compared the effects of ammonia, Glu, TNFα, and NMDA on astrocytic swelling that may result from above mentioned perturbations. The cells swelled to a similar extent upon exposure to each of the four compounds, whereby the cell volume increase induced by ammonia was prevented by MK-801. Obara-Michlewska et al., (2013) [97] showed that 72 h ammonia treatment induces transcription of kynurenine transferase II (KAT II) in rat astrocytes in vitro. The effect was reproduced by the NMDAR antagonist MK-801, indicating that inhibition of NMDAR activity could be involved in the effect of ammonia. The stimulatory effect of ammonia on KATII transcription was mimicked in brains of rats subjected to hyperammonemia due to liver failure, and was potentiated by administration of NMDAR antagonist memantine. Obara-Michlewska et al., (2015) [90] also demonstrated that acute liver failure decreases the expression in the brain of the Kir4.1 channel and that this effect is likewise attenuated by memantine.

## 8. Conclusions and Perspectives

Collectively, the data reviewed in this article demonstrate beyond doubt the presence of NMDARs in the cell membranes of astrocytes. The astrocytic NMDAR is composed of the same set of 7 subunits as is the neuronal NMDAR. Subunit configuration and assembly in astrocytic NMDAR differs somewhat from their composition in neuronal NMDAR, and appears to variate depending upon the source of the astrocytes (species, brain region, degree of maturity). Impact of the subunit configuration on the specific features of the responsiveness of astrocytic NMDAR to external stimulus, such as limited sensitivity to Mg^2+^ blockade, relatively low Ca^2+^ permeability, and the uncertain degree of the dependence of its activity upon the presence of co-ligands (Gly, d-serine), remain to be analysed in more detail. Irrespective of these uncertainties, activation of NMDAR in astrocytes by its natural ligand Glu or NMDA elicits an intracellular response in the form of whole-cell currents and intracellular accumulation of calcium ions. The NMDAR activity may, in different experimental settings, be executed either by the canonical, ionotropic, or the metabotropic mechanism. Since the two mechanisms often concur in one and the same astrocytic preparation, a switch from one to the other may depend upon as yet undefined mechanistic cues. Importantly, the manifestations of NMDAR activity have been documented both in the ex vivo and in vitro settings, rendering the evidence conclusive. 

Relative completeness of the characteristics of the astrocytic NMDAR with regard to its mode of activation and its intracellular manifestations contrasts with the very preliminary character of evidence regarding its contribution to modulation of neurotransmission. To the best of our knowledge, the demonstration that NMDAR-dependent calcium signaling mediates astrocyte-dependent modulation of presynaptic strengths in the CA1 of the hippocampus [88] remains the only data of this kind published so far. It will be of interest to see if and how astrocytic NMDARs are involved in modulating the function of different neuronal circuits in other brain areas. Further studies towards this end will have to account for the emerging evidence that astrocytes in different brain regions differ in their spatial arrangement and the repertoire of active protein moieties [98,99]. 

**Figure 3:**
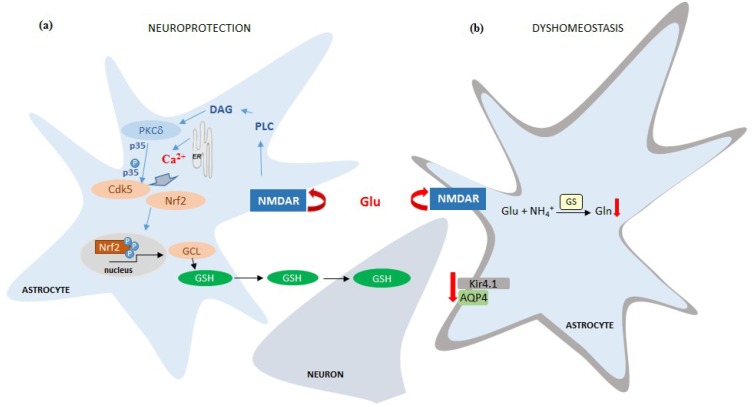
Schematic description of mechanisms by which NMDAR activation in astrocytes leads either to neuronal protection (**a**) or to deregulation of astrocytic proteins critical for their functions (**b**). (**a**) Activation of NMDAR on astrocytes leads to PLC activation triggering the release of Ca^2+^ from the ER. PLC activate PKCδ, likely through diacylglicerol (DAG), leading to p35 phosphorylation, and cyclin-dependent kinase-5 (Cdk5) activation; p35/Cdk5 complex phosphorylates Nrf2 and promotes Nrf2 translocation to the nucleus, inducing the expression of different antioxidant genes, including glutamate-cysteine ligase (GCL), the rate-limiting enzyme of glutathione (GSH) biosynthesis. The increase of GSH synthesis and release supports neurons against ROS-mediated oxidative damage [48]. (**b**) Activation of NMDAR on astrocytes leads to loss of astrocytic proteins critical for their housekeeping functions, namely glutamine synthetase (GS), the water channel protein aquaporin-4 (AQP4), and the inward rectifying potassium channel Kir4.1. (Kir4.1.) [72,90].

Durable extrasynaptic accumulation of Glu is a phenomenon typical of a spectrum of neurological disorders. Recently, a few studies examined the possibility that excessive Glu may—apart from exerting direct neurotoxicity—influence brain homeostasis by interacting with astrocytic NMDARs. Experiments in in vitro models demonstrated that prolonged exposure of astrocytes to NMDA may elicit both a neuroprotective (increased synthesis in astrocytes and subsequent transfer to neurons of the antioxidant GSH) and deleterious response (loss from astrocytes of proteins critical for their housekeeping function) (Figure 3).

In a few instances, reproduction in cultured astrocytes of gliotoxic effects of compounds involved in the pathogenesis of neurodegenerative disorders was likewise mediated by NMDARs. However, functionality of the astrocytic NMDAR will have to be definitively confirmed in the in vivo settings. In the perspective, the use of transgenic mouse models with a conditional knockout of astroglia-located NMDAR subunits may become a useful tool with which to unambiguously demonstrate the involvement of astrocytic NMDAR in the responses of the brain to defined physiological stimuli and pathogens. 

## Abbreviations

β-NGF	Nerve growth factor β
Aβ	Amyloid β
AMPA	α-amino-3-hydroxy-5-methyl-4-isoxazolepropionic acid
AMPAR	AMPA receptors
AP5	2-amino-5-phosphonopentanoate
AQP4	Aquaporin-4
ArchT	Archaerhodopsin proton pump
BMP-4	Bone morphogenetic protein 4
CAPON	Carboxyl-terminal PDZ ligand of nNOS
Cdk5	Cyclin-dependent kinase-5
CNQX	6-cyano-7-nitroquinoxaline-2,3-dione
CNS	Central nervous system
EGFP	Enhanced green fluorescence protein
EPSC	Excitatory postsynaptic currents
ER	Endoplasmic reticulum
FACS	Fluorescence-activated cell sorting
GDNF	Glial cell line-derived neurotrophic factor
Glu	Glutamate
GluN	Subunit of NMDAR
Gly	Glycine
GS	Glutamine synthetase
GSH	Glutathione
IGF-1	Insulin-like growth factor 1
iGluR	Ionotropic glutamate receptors
IL-1β	Interleukin-1β
KA	Kainic acid
KAR	KA receptors
KAT II	Kynurenine transferase II
KYNA	Kynurenic acid
LPS	Lipopolysaccharide
LTD	Long-term depression
L-VGCC	L-type voltage-gated Ca^2+^ channels
MCAo	Middle cerebral artery occlusion
mGluR	Metabotropic glutamate receptors
NMDA	*N*-methyl-d-aspartate
NMDAR	NMDA receptor
NMDARs	NMDA receptors
nNOS	Neuronal nitric oxide synthase
NO	Nitric oxide
NOS1	Nitric oxide synthase 1
Nrf2	Nuclear factor-erythroid 2-related factor-2
OGD	Oxygen-glucose deprivation
PSD95	Postsynaptic density protein 95
QUIN	Quinolinic acid
TBOA	dl-threo-β-benzyloxyaspartate
TCA	Tricarboxylic acid
TTX	Tetradotoxin
